# Robot-Assisted Infratrigonal Vesicovaginal Fistula Repair

**DOI:** 10.1155/2019/2845237

**Published:** 2019-05-26

**Authors:** João Pádua Manzano, Fábio da Silva Crochik, Felipe Guimarães Pugliesi, Renato Vasconcelos Souza de Almeida, Petronio Augusto de Souza Melo, Ricardo Luís Vita Nunes

**Affiliations:** ^1^Surgery Department at Federal University of São Paulo, São Paulo, Brazil; ^2^São Paulo's Military Hospital, Brazilian Army, São Paulo, Brazil; ^3^Division of Urology, Men's Health Centre, Hospital Brigadeiro, Sao Paulo, SP, Brazil; ^4^Benign Prostate Hyperplasia Department in Brazilian Urology Society, Urology Department in Sao Paulo's Military Hospital, Brazilian Army, São Paulo, Brazil

## Abstract

**Background:**

Although relatively rare, vesicovaginal fistula is the most common genitourinary fistula, causing a significant decrease in patients' quality of life. Location of fistula is major supratrigonal, with some cases located in the trigone and rarely below it. Disease treatment is surgical, and repair can be performed by several techniques, including robot-assisted.

**Case Presentation:**

We present a case of a patient who developed an infratrigonal vesicovaginal fistula after treatment of a cervical cancer. The patient was submitted to robotic repair of the vesicovaginal fistula.

**Conclusion:**

The use of robot-assisted laparoscopy is expanding over all areas of urology and its applicability to repair vesicovaginal fistulas brings good results.

## 1. Introduction

Vesicovaginal fistula (VVF) is the most common fistula between the female genital tract and the urinary tract, and it is characterized by drainage of urine through the vagina, with a significant reduction in patients' quality of life [1]. It is presented by urinary flow through the vagina, unrelated to urination, and the volume of loss is directly related to the diameter of the fistula [2, 3]. In low-resourced countries, it often occurs as a result of prolonged obstructed labour due to the ischemia, as the bladder becomes compressed between the foetus and the pubic symphysis. Meanwhile, the VVFs that are seen in well-resourced countries commonly develop following iatrogenic injury, with over 60% following a hysterectomy. In a study of the English National Health Service, one in every 788 hysterectomies is associated with urogenital fistulae [4], occurring about 1 to 6 weeks after hysterectomy, and when recurrent, about 3 months after the first repair [5–8]. In addition, other risk factors also favor the appearance of genitourinary fistulas, such as pelvic surgeries, radiation, infection, and neoplasias affecting the pelvic floor [9, 10]. The most common location of the fistulas is supratrigonal, with fewer cases of trigonal and infratrigonal fistulas [11–13].

Investigation of the disease should always contain a detailed pelvic evaluation, with specular examination of the vagina and cystoscopy [10]. The vaginal tamponade test with infusion of intravesical methylene blue can also be performed but only serves to identify the presence of the fistula, without assessing size, position, number, and complexity [9]. Other exams such as cystography and micturition urethrocystography may help in the evaluation of the fistula. Another important point is always to evaluate the upper urinary tract, since concomitant ureteral lesions are present in about 12% of the cases, and computed tomography with intravenous contrast or even a pyelography may be performed during cystoscopy [14].

## 2. Case Report

**Figure 1:**
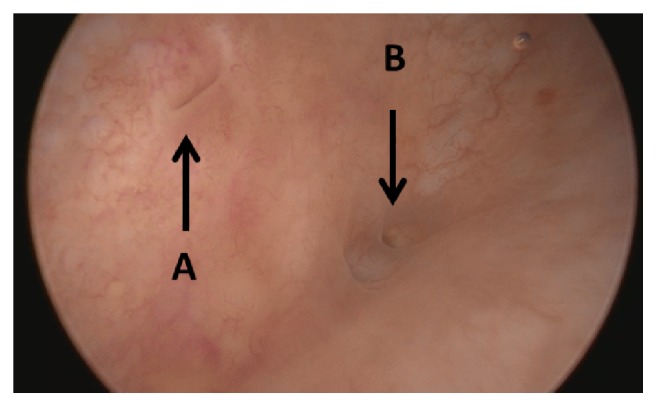
Cystoscopy performed before surgery. A: left ureteral meatus; B: fistulous orifice.

L.O., a 58-year-old female married white patient, with previous history of subtotal hysterectomy in 2012 due to endometriosis, was diagnosed in 2016 with invasive endocervical adenocarcinoma, being treated with colpectomy and brachytherapy. During follow-up, progression of the disease was detected, with metastases in the liver, the peritoneum, and the vaginal dome. In 2017, she was submitted to the excision of the peritoneal implants, the hepatic lesion, the omentum, the vaginal dome, the tuba, and the left ovary. Pathological analysis confirmed metastatic lesions in the vaginal dome and peritoneum, without neoplasia in the other resected tissues. She was submitted to adjuvant chemotherapy with carboplatin and paclitaxel weekly and bevacizumab every 21 days. About 2 weeks after the last surgery she complained of moderate amount of continuous urinary loss through the vagina and the use of 3 to 4 PADs per day. Despite the continuous loss, she continued to urinate through the urethra. Urinary urgency episodes were also reported, with no response to oxybutynin and mirabegron. Recurrent urinary tract infection was not present. A complete evaluation was performed with specular examination, urethrocystography, and contrasted computed tomography, with no lesions identified. Cystoscopy was then performed and revealed a 3mm diameter infratrigonal fistulous lesion, right under the left meatus (Figure 1).

**Figure 2:**
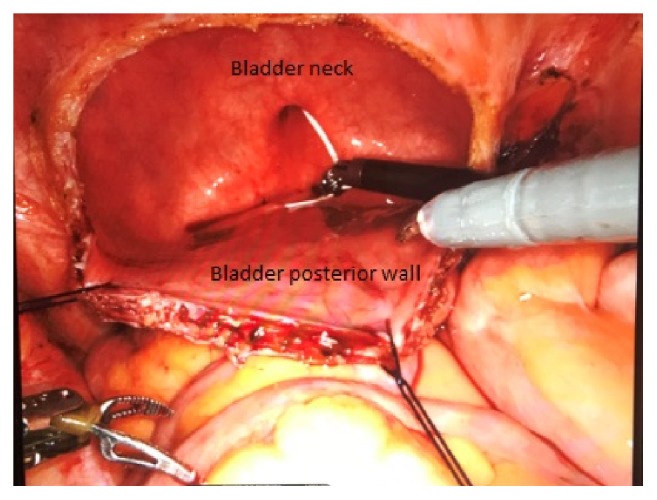
Transperitoneal view of open bladder: a white ureteral catheter positioned through urethra into left ureteral meatus.

**Figure 3:**
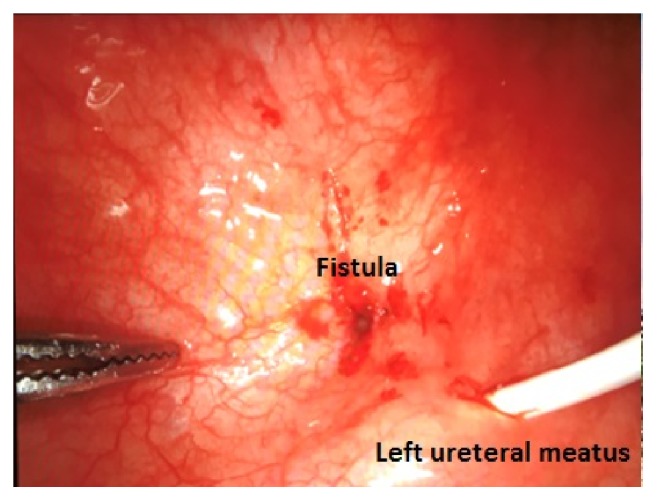
Fistula identification: in the transperitoneal view infratrigonal fistula is identified above the ureteral meatus.

**Figure 4:**
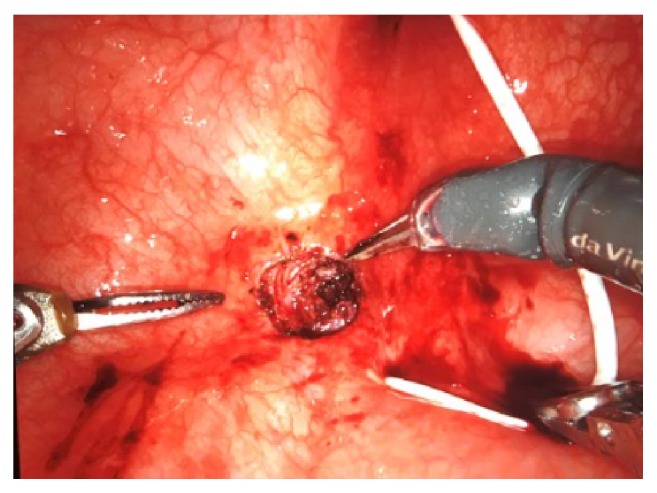
Fistula dissection is performed by separating vaginal and bladder sides.

**Figure 5:**
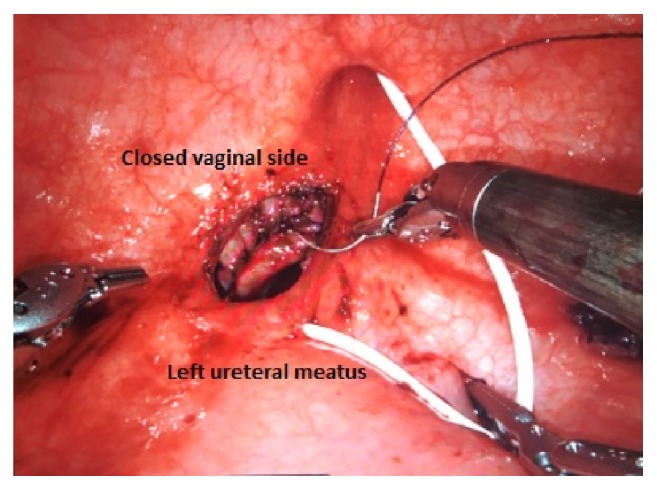
Vaginal side sutured by 3-0 barbed suture in 2 layers.

**Figure 6:**
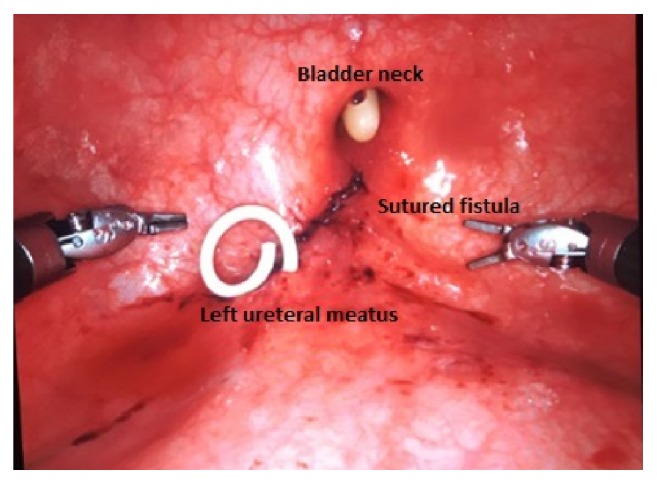
Final appearance of the treated fistula after suture of bladder.

Patient underwent robot-assisted repair of the vesicovaginal fistula, with transperitoneal access. First, the patient was positioned in lithotomy and a cystoscopy was performed, identifying the fistulous orifice right under the left ureteral meatus. An ureteral catheter was placed thought the urethra in the left ureter. The position was then changed to a steep Trendelemburg and 5 ports were inserted: one 12mm optic port (3cm above the umbilicus and 1cm left of the middle line), three 8mm robotic ports (at the umbilicus level, symmetrically placed 2 ports on left and right pararectal line, and one more port placed up from the iliac crest on the left side), and one 5mm assistant port (placed up from the iliac crest of the right side). After the ports were placed, the robot was docked and the laparoscopy initiated. Right at the beginning of the laparoscopy, a lot of adherences were visualized, needing a careful adhesiolysis of the bowel from the surrounding structures. With the bladder well dissected, a transversal cystotomy was performed, to expose the vesical side of the fistula (Figures 2 and 3). The fistula was dissected with a good margin of healthy tissue until vaginal side (Figure 4). The synthesis was initiated with a barbed 3-0 continuous suture (V-Loc™), closing the vagina. The vesical side was closed in 2 layers, using the same suture (Figure 5). In the end of procedure, a 4.7mm ureteral stent and an 18Fr bladder catheter were placed (Figure 6). The bladder was also closed with the 3-0 barbed suture (V-Loc™). Total operative time was 87 minutes, estimated blood loss was less than 50mL, and the length of hospitalization was 30 hours. Bladder catheter remained for 2 weeks and the ureteral stent for 4 weeks. After the withdrawal of bladder catheter, patient remained well, without further complaints and no longer losing urine.

## 3. Discussion

There are several ways to treat vesicovaginal fistulas, including conservative treatment with 8% of success [15]. Given this poor result of nonoperative treatment, surgery is the main way of treatment. The most important principle in repair is to provide a tension-free, watertight closure, and the surgical route should be the one that provides the best possible chance of closure on the first attempt [16]. There are different ways of repairing a vesicovaginal fistula including vaginal, abdominal, and laparoscopic/ robotic approaches [17]. The route depends partly on the characteristics of the fistula but also on the experience of the surgeon. Two conventional approaches for VVF repair are transabdominal repair for supratrigonal VVF and transvaginal approach for low lying fistulae [18]. An ideal time for repair is still debatable. Irrespective of the approach used the principle of VVF repair remains the same, i.e., the separation of bladder and vagina, closure of the fistula in 2 separate layers preferably perpendicular to each other, tissue interposition, and urinary drainage [19].

The first fistula treated by open abdominal access was described in 1803 [20]. In the 1990s, with evolution of technology and the aim of reducing surgical morbidity, laparoscopic approach was described for the first time [21]. Despite the minor trauma, the laparoscopic technique did not initially have as many supporters probably due to the technical difficulty of dissection of vesicovaginal fistula and intracorporeal suture. Given this difficulty, Melamud et al. described for the first time in 2005 the robotic correction of a vesicovaginal fistula, showing good results and less difficulty in dissection and suturing with the articulated arms [22]. Since that moment the robotic technique is increasing, with good results even in complex cases [23].

In a comparative study between open and robot-assisted repair of recurrent supratrigonal VVF, Gupta et al. demonstrated similar efficacy but significantly lower morbidity in terms of blood loss and postoperative hospital stay with the robot-assisted approach [24]. The advantages of using the robotic system for this surgery are evident from the outset as it needs reconstruction deep inside the pelvis [25]. However, data on the use of robotic-assisted approach in managing VVF is still limited.

As reported in the literature, the operative times vary between 95 minutes and 305 minutes. This heterogeneity arises from varying surgeon experience and variability in timings itself as few authors reporting only the console time. The blood loss is usually insignificant varying between minimal and 120 ml. The length of hospitalization is usually short, in consistence with the prevalent advantages of minimally invasive approach. The mean follow-up period is also variable between 3 months and 28.3 months after surgery [26]. The data we report are compatible with literature.

Several reports describe the repair of supratrigonal fistulas but there are few descriptions of infratrigonal repairs with robot-assisted technique, as the location of fistula favors transvaginal repair. Furthermore, as the patient had a previous omentectomy, no tissue was interposed between the two layers of the suture. Various interposition flaps have been described in the literature, including omental flaps, peritoneal flaps, and amniotic allograft interposition tissue flaps. An interposition flap for VVF works on two theoretical principles: it functions as a barrier and it introduces vascular and theoretically lymphatic vessels that improve tissue growth and maturation. Omentum is the most common flap described in literature. In the absence of endogenous tissue, the use of biological sealants has also been reported (e.g., Fibrin glue) with the aim of avoiding fistula relapse and showing good results [22, 26]. Our case shows the possibility of performing robot-assisted transabdominal repair of infratrigonal fistulas without interposed tissue, with no more urine loss complaints after one year of follow-up.
